# A correlation study of *BK Polyoma Virus* infection *and* prostate Cancer among Sudanese patients - immunofluorescence and molecular based case-control study

**DOI:** 10.1186/s13027-019-0244-7

**Published:** 2019-09-18

**Authors:** Babbiker Mohammed Taher Gorish, Mohammed Elfatih Hussein Ournasseir, Iman Mohammed Shammat

**Affiliations:** 1 Department of Microbiology and Immunology, Faculty of Medical Laboratory Science, Omdurman Islamic University, Omdurman, Sudan; 2 Department of Biology, Faculty of Science, Taibah University, Medina, Saudi Arabia

**Keywords:** Prostate cancer, Benign prostatic hyperplasia, *BKV*, Large T antigen, Immunofluorescence, PCR, Q-PC, Real-time PCR

## Abstract

**Background:**

*Polyomavirus hominis1*, also called *BK virus* (*BKV*) is a well-known etiological agent of renal transplant nephropathy and cystitis. Recently, it got great attention from the researcher as a principal predisposing factor for different kinds of cancers including prostate cancer (PCa). Thus, this study aims to determine the correlation between *BKV* infection and PCa through a descriptive case-control based study.

**Methods:**

A total of 55 paraffin-embedded tissue blocks of patients with PCa and another 55 tissue blocks from BPH patients were obtained. In parallel, respective urine samples were collected from all the cases and controls. The existence of *BKV* large T antigen (LTAg) was analyzed by Direct Immunofluorescence assay. Only *BKV* LTAg positive specimens were further analyzed for the presence of viral DNA by using a conventional PCR then subjected to viral load quantitation by using Q-PCR.

**Result:**

*BKV* LTAg was identified in 30% (17/55) of cases tissue specimens and only in 7% (4/55) of the controls tissue specimens with *P-*value 0.002 and Odd ratio 5.7. The conventional PCR detects the *BKV* DNA in 16 out of 17 cases specimens while only two out of four controls specimens were identified with a viral DNA. The mean of the *BKV* DNA load was higher significantly among cases 6733 ± 6745 copies/ml when compared to controls 509.0 ± 792.9 copies/m with a *p*-value of 0.002.

**Conclusion:**

More *BKV* prevalence with high viral load was observed in PCa patients tissue compared to BPH specimens. PCa Gleason scores 9 and 7 were the most cancer grades identified with the presence of *BKV* DNA. Our findings are thus consistent with a significant link between the *BKV* infection and the PCa risk. Prostate or seminal fluids should be selected as principal specimens for future studies and can, therefore, be designated as screening samples to find early virus evidence in the prostate tissue. Detection of early virus evidence may help to reduce the risk of PCa cancer due to *BKV.*

## Background

The prostate is one of the most essential males’ exocrine glands. It is prone to various pathological conditions in which malignant and benign diseases are the most common [1]. The Benign Prostatic Hyperplasia (BPH) which is not cancer and familiar among older men occurs when the prostate gland turns to be larger than normal size and when the gland becomes larger it can ‘squeeze’ the urethra and this will display several manifestations such as difficulty urinating and frequent needs to urinate during the day [2].

Prostate cancer (PCa) is a biologically homogenous tumor that is one of the leading causes of cancer deaths in men [3]. The GLOBOCAN 2018 report estimated that there would be approximately 1.3 million new prostate cancer cases and 359,000 related deaths globally in 2018, which led to PCa being classified as the second most frequent cancer and the fifth leading cause of cancer deaths among men [4]. Also, the report demonstrated that about two-thirds of the newly diagnosed cases will be determined in the countries with very high developing index, where only 18% of the world’s male population resides [5, 6].

Throughout the past four decades, the incidence rates of PCa are increased dramatically in many countries at various development levels. In GLOBOCAN 2018 report the incidence rates of PCa vary by more than 100-fold worldwide and are highest in some Caribbean islands, Australia/New Zealand, Northern and Western Europe, and Northern America, and lowest in Asia. The disease is considered as a leading cause of cancer death among men in 46 countries, particularly in Sub-Saharan Africa and the Caribbean [4, 5, 7]. The death rates due to prostate cancer have been decreasing in many countries, including those in Northern America, Oceania, Northern, and Western Europe, developed countries of Asia, and The United States, on the contrary, there is a rising in mortality rates in several Central and South American, Asian and Central and Eastern European countries, including Cuba, Brazil, the Philippines, Singapore, Bulgaria, Belarus, and Russia, [4, 5].

In Africa, the GLOBOCAN 2018 report determined that the incidence rates of PCa per 100,000 populations ranged from 66.9–111.8 in Southern Africa to less than 16.3 per 100,000 populations in Northern Africa countries such as Egypt, Libya Algeria and in some middle Africa countries such as Sudan. Also, the GLOBOCAN 2018 report stated that the mortality rate due to PCa per 100,000 population in Africa ranged from 24.4 in southern Africa to 18.7 in Eastern Africa with least mortality rate of 7.0 determined in Northern Africa [5, 8, 9].

In Sudan, PCa is now recognized as one of the principal medical problems facing the male populations and according to the report form, Radiation and Isotopes Center of Khartoum (RICK) PCa is the most common cancer in Sudanese males (3.3%) [8]. The incidence of prostate cancer has increased dramatically in the past 20 years and the disease has gained increased attention from Sudanese urologists [10]. Moreover, about 600 Sudanese men have diagnosed with PCa annually [1], and mortality rates are about 8.7 per 100,000 populations. The disease was found equally distributed among different tribes and most cases (85.4%) presented with stage III and IV [9].

PCa is generally, a slow-growing and the majority of men can live with it for a long time without painful symptoms or spread. Early PCa usually causes no symptoms. However, prostate cancer causes symptoms often similar to those of diseases such as BPH. In the early stage of prostate cancer, there are usually no symptoms, but later stages can cause symptoms that include frequent or sudden need to urinate, difficulty to urinate, blood in urine and pain in various bones if cancer has spread to them. Obstructive symptoms occur at the clinical metastatic castration-resistant status due to the tumor bulk. Also, bone-pain is caused by bone-metastatisation [2].

There are several risk factors to develop prostate cancer and these include ages, races, ethnicity, alcohol consumption, genetic factors, farmers, a diet high in fat, tire plant workers and men who be around cadmium in addition to infections with certain viruses [10–15].

The human *BK polyomavirus is* a member of the *polyomavirus* family and it is small non-enveloped icosahedral DNA virus, the capsid encloses a circular double-stranded DNA genome of approximately 5100 nucleotides that is coated by host-cell histones. It was first isolated from the urine of a renal transplant patient [16]. The virus infects almost 90% of the human population worldwide. It resides in a subclinical persistent state in the urinary tracts of healthy individuals and reactivates in immunosuppressed transplant patients, in whom it is associated with hemorrhagic cystitis and *polyomavirus* nephropathy [17]. Also, the urinary shedding has been reported to occur asymptomatically and intermittently in healthy individuals [18].

Based on serological and genotyping techniques *BKV* has been categorized into four subtypes [19]. Subtype I is the most dominant one and have world-wide distribution; subtype IV is the coming directly after subtype I and mostly isolated from East Asia. Although subtypes II and III are isolated worldwide, their frequencies are low [20]. According to the phylogenic investigations subtype I itself is divided into 4 subclasses including subgroups 1/a, 1/b-1, 1/b2 and 1/c; each one is distributed in a certain geographical location. While subtype IV, is subdivided into six subclasses including 4/a-1, 4/a-2, 4/b-1, 4/b-2, 4/c-1 and 4/c-2 [19].

The genome of *BKV* is divided into early, late and regulatory regions (NCCR). It encodes for at least six proteins, two from the early region and four from the late region. The early proteins include the large tumor antigen (LTAg) and the small tumor antigen (STAg). The LTAg promotes cellular transformation by interfering with the tumor suppressor functions of p53 [21]. Whereas the STAg induces tumorigenesis and promotes anchorage-independent growth of transformed cells by the negative regulation of protein phosphatase 2A [22, 23].

The Underlying causes of PCa are not completely understood, but it may likely occur due to a combination of factors such as aging, family history and dietary factors in addition to infectious agents [23]. Recent investigations supported a possible carcinogenic activity of the human *BK polyomavirus* in the prostate tissue [23]. And this may be due to their large and small T antigen. In addition, in vitro studies supported the oncogenic contribution of the large Tag and small tag with the potential to cooperate with other oncogenic alterations. Despite the suggestive mechanistic evidence, the role of *BKV* in human malignancies is controversial [18].

In Sudan, only few researchers have addressed the subject under study and most previous work have only focused on the genetic factor. Hence, our study attempted to determine if the *BKV* infection is associated with prostate tumors and, if so, whether viral oncogenes are expressed. To accomplish this, tissues from PCa and BPH individual patients were analyzed by using immunological and molecular technique.

Our investigation differs from previous analyses in the application of the IF test to detect antigen which is responsible for the carcinogenesis and real-time PCR whereas most of the previous studies were using a conventional PCR only to analyze the hypothesis. Also, we include a large sample size in comparison to previous studies.

## Methods

### Study area

This study was a descriptive case control based study that carried out in three hospitals in Khartoum State: Police’s Hospital, Military Hospital, and Soba University Hospital. The laboratory investigations were done in the Central Research Laboratory and Research Laboratory of Veterinary College-Bahri University, Khartoum. The study was conducted during the period from September 2017 to November 2018.

### Study population

Sudanese patients histologically confirmed with PCa were selected as cases group. While BPH patients who had no evidence of cancer were selected as a control group. All cases and controls were newly diagnosed and not selected from hospital registration and they were not under any kind of treatment during samples collection. The diagnosis of PCa and BPH was performed based on the evaluation of the prostate tissue biopsy and ultrasound images, radionuclide scintigraphy, and MRI studies. After staining the slides by H&E staining method it was examined by a well-expertized pathologist who confirmed the PCa diagnosis for cases samples and BPH diagnosis for controls samples. The controls group was matched with cases group in the age, gender, socioeconomic status, geographical factors, environmental factor and, the tribal affiliation. A total of 110 Formalin-fixed prostate tissue biopsies were obtained for both cases and controls (55 each). In parallel, 50 ml of urine samples were collected in a clean, dry universal container from each respective case and control subject. The samples were stored at − 20 until analysis.

### Inclusion and exclusion criteria

#### Inclusion criteria

Based on prostate tissue biopsy evaluation and ultrasound images, radionuclide scintigraphy, and MRI studies those patients who were confirmed to have PCa (in cases) and BPH (in control) were included only in this study. No restrictions were placed based on tribal affiliation or age. Only newly diagnosed patients were included in our study.

#### Exclusion criteria

Patients with prostate tissue Atypia, or have a history of hemorrhagic cystitis disease due to *BKV* or *have a history of polyomavirus* nephropathy disease were excluded from this study.

### Data collection

We used an interviewer-administered questionnaire to ask cases and controls about their demographic, socioeconomic, and geographical afflation, cadmium contact, alcohol consumption, as well as clinical data (including grade and family history of prostate cancer in addition to the duration of the early patients symptoms recognition (DoPESR) which we were defined by the time from the early patients symptoms onset to the time at which his confirmatory diagnosis was made in the hospital to which he was referred). Laboratory investigation data were also recorded. Prior to commencing the study, the proposal was ethically approved by the ethical committee of Omdurman Islamic University. Then, informed consent from each patient and permission from the general managers of hospitals was obtained**.**

### Direct immunofluorescence assay

Immunofluorescence assay was carried out using a primary antibody conjugated to a fluorophore (*Polyomavirus* large T antigen Antibody (PyLT) Alexa Fluor® 488 code (sc-53,479 AF488) supplied Santa Cruz Biotechnology, Inc. U.S.A). And all steps including Section preparation, antigen retrieval, section staining, and examination were done according to formalin-fixed, paraffin-embedded Santa Cruz Biotechnology direct immune fluorescence assay protocol [24]. Then, immediately the specimens were mounted with mounting medium and by fluorescent microscope within 30–60 min after staining. Then the slides were kept in a dark box at 4 °C for storage. Positive (urine epithelial cells with *BK Virus)* and Negative control (normal skin tissue sample) were included to confirm reagent stability and to exclude false positive or false negative result. Moreover, Bancroft and Gamble standard guidelines of histological techniques were used In order to reduce the risk of tissue contaminants during sectioning and staining [25].

### Molecular analysis and examination

Only biopsies of prostate tissue and urine samples that were revealed a positive *BKV* LTAg IF reaction were examined for the *BK* viral DNA by using a conventional PCR and real-time PCR techniques.

### DNA extraction

About 25 mg of each tissue sample was incubated in lysis buffer (20 μl proteinase K (200 mg/ml) and 5 μl of RNAase A). The DNA extraction was performed by the DNeasy® Tissue Kit (QIAGEN Company) according to the manufacturer’s instructions and stored at − 80 °C until further analysis. The quality of DNA was checked using Nano drops test. In the other hand, one ml of the urine sample was incubated in lysis buffer (20 μl proteinase K (200 mg/ml) and 5 μl of RNAase A). The DNA extraction was performed by the DNeasy® Urine Kit (QIAGEN Company) following the manufacturer’s instructions and stored at − 80 °C until further analysis.

### Conventional (standard) PCR analysis

*BK* viral early gene region (Large T antigen region176bp) was amplified in a master mix reaction volume of 25 μl containing 5 μl of DNA sample and specific forward and reverse primer 1 μl for (primer F: 5′-AGTCTTTAGGGTCTTCTACC-3′ and *BK*127-R: 5′-GGTGCCAACCTATGGAACAG-3′) [26] and completed to 25 μl with 13 μl Distilled water. The amplification was done by using (TC-3000 conventional PCR Thermal Cycler, USA) through 40 cycles of denaturation at 94 °C for 1 min proceeded by initial denaturation at 94 °C for 5 min, annealing at 55 °C for 1 min and extinction at 72 °C for 2 min followed by a final extension at the same temperature for 5 min. Finally, the conventional PCR products were subjected to the gel electrophoresis (2% agarose gels) to visualize the band of the amplified target gene region with ethidium bromide (0.5 μg/mL) for 30 min in a UV-gel documentation system. Positive and Negative control was included in each PCR assay.

### Quantitation of the *BK polyomavirus* load

Q-PCR assay was performed by using Rotor-Gene Q (QIAGEN, Germany) machine. Real-time PCR amplification was done following the manufacturing procedure (*Polyomavirus BK* Real-Time PCR Kit, Shanghai ZJ Bio-Tech Co., Ltd) in a reaction volume of 40.4 μl containing 4 μl of DNA sample, standards and negative control, 34μlof PBK Reaction Mix, 1μlQ-Amplimix (forward and reverse primers) specific for *BK* virus (primer F: 5′-GCA GCT CCC AAA AAG CCA AA-3′ and BK127-R: 5′-CTG GGT TTA GGA AGCATT CTA-3′) in addition to 1 μl Internal control and 0.4 μl of Q-PCR Enzyme Mix (hydrolysis probes5′-AGCTGGAACACAACAGTGGAGAGGCC-3′). Thermal cycling was initiated with a first denaturation step of 10 min at 95 °C, followed by 40 cycles of 93 °C for 15 s and 60 °C for 1 min. The detection of amplified *Polyomavirus BK* DNA fragment quantities was performed in fluorimeter channel FAM with the fluorescent quencher BHQ1. Furthermore, the possible PCR inhibition was identified by measuring the HEX/VIC/JOE fluorescence of the internal control (IC). An external positive control (1 × 107 copies/ml) was diluted by distilled water to allow the determination of the gene load. The amplification data were analyzed with Rotor-Gene Q software. The number of BK virus copies in each sample was calculated from the standard curve.

### Quality control

Samples were collected, transported and stored appropriately to obtain good results. Proper installation, tissue processing and blocking steps were applied during the IF test. Positive (epithelial urine cells with BK virus) and negative control (normal tissue skin sample) were also incorporated during the IF test. To confirm the stability of the reagents during the conventional PCR assay, we include a purified BKV gene from the Dunlop strain as a positive control and distilled water as a negative control. Briefly, amplification of beta-globin was done for all samples to control DNA extraction. All experiments were done in a contamination-free environment. Standard precautions are designed to prevent contamination during Q-PCR. Possible Q-PCR assay inhibition was identified by measuring the HEX/VIC/JOE fluorescence of the internal control (IC). The kit of Q-PCR assay was provided a ready to use positive and negative controls which were included in each Q-PCR assay.

### Statistical analysis

Statistical analysis was performed using SPSS version 20 (Statistical Package for the Social Sciences). *P*-value is significant at a level equal to or less than 0.05. And the result was presented through various graphics and tabulated modules. Also, a special statistical test such as chi-square was performed to demonstrate the ability of *BK virus* to develop carcinogenic changes in prostate tissue.

## Result

### Detection of large T antigen in the prostate tissue specimens

**Fig. 1:**
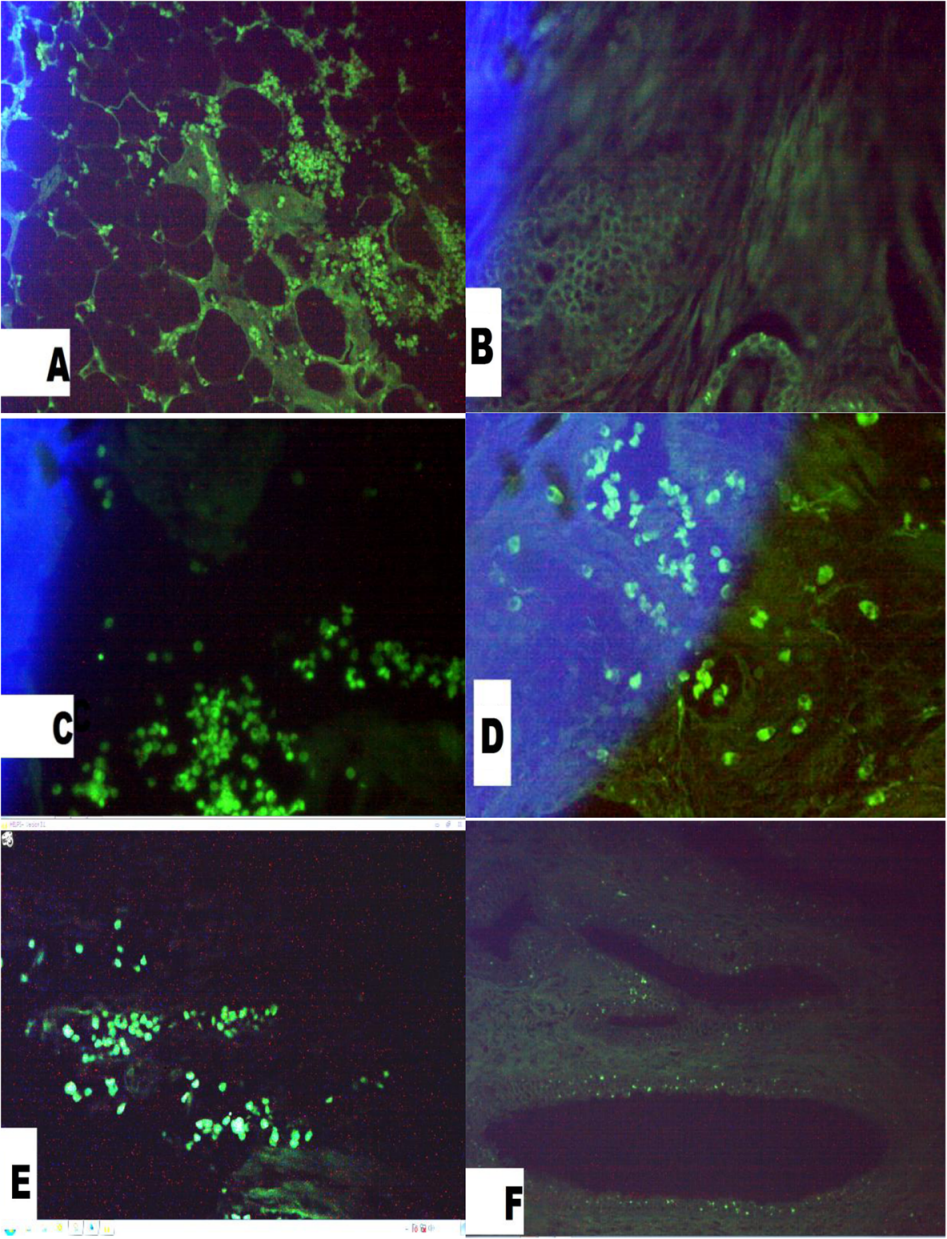
Detection of BKV large T-antigen expression by direct immunofluorescence assay. **a, c** sections of the prostate cancer tissue show a positive IF reaction 10X magnification. **d** prostate cancer section shows a positive IF reaction by 40X magnification. **b** section from PCa patient shows a negative IF reaction 10X magnification. **e** section from BPH patient shows a positive reaction 10X magnification. **f** section from BPH patient shows a negative IF reaction 10X magnification

Immunofluorescence assay showed that the *BKV* LTAg was positive in 21 (19%) prostate tissue specimens out of 110 samples that were examined (55 cases and 55 controls). The *BKV* LTAg prevalence among cases was 30.9% (17/55) and was higher than that among controls 7.2% (4/55). This variation in the prevalence of LTAg between cases and controls was analyzed by Chi-square test and found to be a highly significant with *P*-value 0.002 and odd ratio 5.7 (Fig. 1), (Table 1).

**Table 1:** Shows the prevalence of the BKV Large T antigen among cases and controls (samples were examined using Immunofluorescence assay) along with risk estimation among cases group

Participants	Result of IF test		Total	Odd ratio For patients
	Positive	Negative		
Result of IF test				
Case	17 (30.9%)	38 (69.1%)	55	
Control	4 (7.2%)	51 (92.3%)	55	5.704
Total	21	89	110	

The study compared the age and the DoPESR in only *BKV* Large T antigen positive *(BKV LTAg+*) cases and controls and found that cases showed an average age of 70.71 ± 7.3 years which is a higher than those in controls group 67.25 ± 3.4 years. This difference is statistically insignificant since the *P*-value was 0.380 (Table 2). While the mean of the DoPESR was significantly lower in cases 8.29 ± 2.3 than that in the controls group 11.50 ± 3.0 months with *P*-value of 0.03 (Table 2).

**Table 2:** Shows the mean of age, DoPESR and Viral Load mean among cases with BKV LTAg+ compared to controls with BKV LTAg+ as well as the mean of age and DoPESR for cases only comparing those that are BKV LTAg+ vs BKV LTAg. The *P*-value is significant at the level equal to or less than 0.05

Parameter	Participants	N	Mean	Std. deviation	*P*-value
Age/years	Cases with *BKV LTAg+*	17	70.71	7.389	0.380
	Controls with *BKV LTAg+*	4	67.25	3.403	
	*Cases with BKV LTAg+*	17	71.82	7.469	0.582
	*Cases with BKV LTAg-*	38	70.61	7.561	
DoPESR / months	Cases with *BKV LTAg+*	17	8.29	2.3	0.03
	Controls with *BKV LTAg+*	4	11.5	3.0	
	*Cases with BKV LTAg+*	17	7.88	2.5	0.271
	*Cases with BKV LTAg-*	38	8.7	2.6	
Viral load copies/ ml	Cases with *BKV LTAg+*	17	6733.71	6745.396	0.002
	Controls with *BKV LTAg+*	4	509.00	792.997	

The 55 cases were categorized into two groups; one group included 17 cases with *BKV LTAg +* and another group with 38 cases with a negative large T antigen reaction *(BKV LTAg-)*. Cases with *BKV LTAg +* showed an age mean of 71.8 ± 7.4 years which is higher than those in *BKV LTAg-* group 70.6 ± 7.5 years but the difference was remained statistically insignificant since the *P*-value was 0.58. Moreover, the cases with *BKV LTAg +* were showed a mean of DoPESR of 7.88 ± 2.5 months which is lower than those with *BKV LTAg-* group 8.7 ± 2.6 months again here the difference is statistically insignificant since the *P*-value was 0.271 (Table 2).

In the PCa cases group, the result showed that *BKV LTAg +* was more frequently seen in patients over the age of 65 years than those under the age of 65 years, but the variation was statistically insignificant with *P*-value of 0.420. On the other hand, when we categorized the DoPESR in two groups, we found that *BKV LTAg +* was seen more frequently among a group of patients with less than 9 months of symptoms recognition. While those who have more than 9 months of symptoms recognition the *BKV LTAg +* was seen less frequently and also here the difference between the two groups was not statistically significant with P-value of 0.321(Table 3**)*.***

**Table 3:** Shows a comparison between cases age groups as well as the DoPESR groups based on BKV IF test results and DNA Viral load means. P-value is significant at a level equal to or less than 0.05

Parameter	Participants groups	Result of IF		P-value	Mean of the Viral load for cases copies/ml	P-value
		Positive	Negative			
Age/years	Less than 65	4	11	**0.420**	6786.6	0.561
	More than 65	13	27		6711.7	
DoPESR / month	Less than 9 month	14	25	**0.325**	4949	0.045
	More than 9 month	3	13		12,535	

Among cases, the highest prevalence of *BKV* large T antigen was identified among patients from central Sudan followed by Southern, Northern, Eastern, and Western Sudanese with a percentage of 59.1, 23.5, 5.8, 5.8, and 5.8% respectively.

Both PCa Gleason scores 9 (4 + 5) and 7 (3 + 4) were significantly associated with the highest prevalence of *BKV* large T antigen, followed by score 6 (3 + 3), 8(4 + 4) and 10 (5 + 5) with a percentage of 29.4, 29.4, 17.7, 17.7, and 5.8% respectively with *p*-value of 0.045 (Table 4).

**Table 4:** Shows a comparison between cases Gleason score grades in the infectivity rate. P-value is significant at a level equal to or less than 0.05

Result of IF test	Cancer Grade (Gleason score)					Total	*P*-value
	3 + 4	3 + 3	4 + 4	4 + 5	5 + 5		
Positive	5 (29.4%)	3 (17.7%)	3 (17.7%)	5 (29.4%)	1 (5.8%)	17	
Negative	14 (36.8%)	9 (23.7%)	7 (18.4%)	6 (15.8%)	2 (5.3%)	38	**0.045**
Total	19	12	10	11	3	55	

The correlation between some PCa risk factors and the prevalence of *BKV* Large T antigen was also determined in the cases group. The result found that there was statistically insignificant role of alcohol consumptions, working in farmer, taking a diet high in fat, working in a tire plant, and family history of cancer and cadmium contact in the prevalence of *BKV* Large T antigen among prostate cancer cases group (Table 5).

**Table 5:** Describe the role of PC risk factors in developing PCa due to BKV infection. A comparison between the BKV LTAg+ and BKV LTAg- cases in the presence or absence of the PC risk factors. P-value is significant at a level equal to or less than 0.05

Result of IF test	Count		
	Alcohol consumption		*P*-value
	Yes	NO	
Positive	6 (35.3%)	11 (64.7%)	**0.106**
Negative	6 (15.7%)	32 (84.3%)	
	Working in farmer		*P*-value
	Yes	No	
Positive	7 (41.1%)	10 (58.9%)	**0.620**
Negative	13 (34.2%)	25 (65.8%)	
	Diet high in Fat		*P*-value
	Yes	No	
Positive	8 (47.0%)	9 (53.0%)	**0.05**
Negative	8 (21.0%)	30 (79.0%)	
	Tire Plant Working		*P*-value
	Yes	No	
Positive	3 (17.6%)	14 (82.4%)	**0.284**
Negative	3 (7.9%)	35 (92.1%)	
	Family history of the disease		*P*-value
	Yes	No	
Positive	5 (29.4%)	12 (70.6%)	**0.371**
Negative	16 (42.1%)	22 (57.9%)	
	Cadmium contact		*P*-value
	Yes	No	
Positive	2 (11.8%)	15 (88.2%)	**0.0696**
Negative	6 (15.8%)	32 (84.2%)	

### PCR and quantitative PCR

A total of 21 samples of prostate tissue and respective urine samples of patients with *BKV* LTAg + (17 of cases and 4 of controls) were subjected to conventional PCR and real-time quantitation PCR tests.

### Conventional PCR

**Fig. 2:**
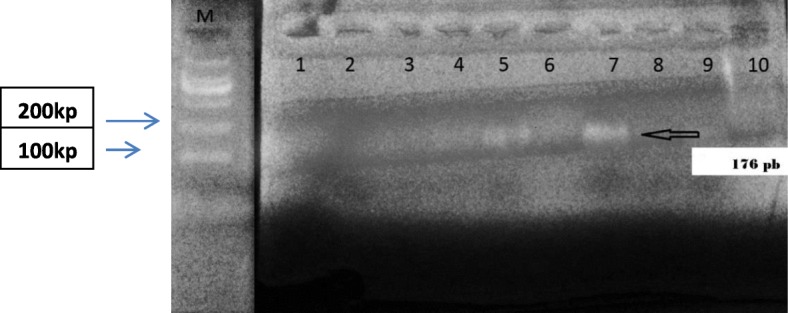
Conventional PCR Result. The products visualized by electrophoresis on ethidium bromide-stained 2% agarose gel. Lane M: low molecular weight marker DNA (100 bp MBI Fermentas) lane 1 contains the negative control, Lane 7 contains the BKV positive control. Lanes 2, 3, 4 and 6 were negative samples, lane 5 shows a BKV positive band (176 bp)

The result of the conventional PCR test confirmed the presence of *BKV* DNA in 18 (86%) out of 21 tissue samples of which 16 specimens (89%) were obtained from the cases and only 2 (11%) were taken from the controls subjects with *P*-value of 0.023 which means that there was a significant difference between cases and control in the prevalence of the *BKV* DNA (Fig. 2), (Table 6). On the other hand, only 4 urine samples out of 21 were confirmed for the presence of *BKV* DNA by conventional PCR of which 3out of 4 (75%) were obtained from controls subjects and only one out 17 samples (5.8%) was obtained from the cases subjects with *P*-value of 0.002 which reflect a highly significant difference between the cases and controls urine samples in the prevalence of the *BKV* (Table 6).

**Table 6:** Shows a comparison between cases and controls based on the prevalence of BKV DNA in their tissue and urine samples (the specimens were examined by using a conventional PCR). P-value is significant at a level equal to or less than 0.05

Type of patients		PCR Result		Total	*P*-value
		Positive	Negative		
Tissue sample	Case	16 (94%)	1 (6%)	17 (100%)	**0.023**
	Control	2 (50%)	2 (50%)	4 (100%)	
Total		18	3	21	
Urine sample	Case	1 (5.8%)	16 (94.2%)	17 (100%)	**0.002**
	Control	3 (75%)	1 (25%)	4 (100%)	
Total		4	17	21	

### Quantitative PCR

**Fig. 3:**
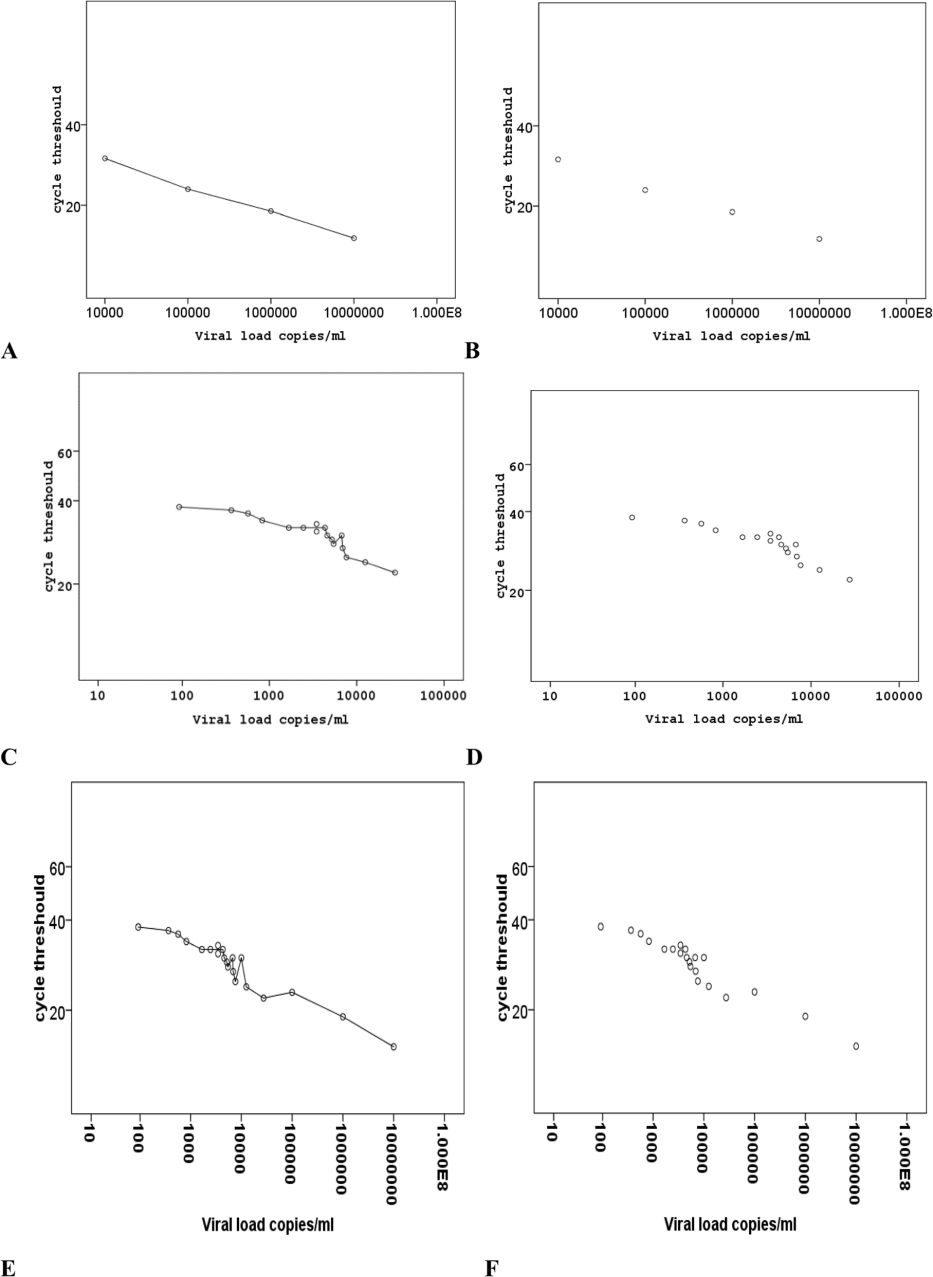
Scatter graph of Q- Real time PCR. **a**, **b** show Standard curve for BKV rt-QPCR. BKV plasmid DNA, in serial dilutions ranging from 10,000 to 10,000,000 copies/ml, has been amplified by rt-Q PCR. Fluorescence intensity was plotted against cycle number. **c**, **d** describe the distribution of BKV loads expressed as DNA copies per milliliter. Short lines indicate medians and ranges of the viral load in the samples. The highest viral load was 27,421 while the lowest viral load of 92 copies/ml**. e**, **f** combined scatter diagram between the samples curve and the standard curve. The Linear range of the assay obtained by use of known serial dilutions of the BKV plasmid as a template. The BKV copies number per samples were calculated from the standard curve

The mean of the viral load of all 21-prostate tissue (cases and controls collectively) samples was 5548.05 ± 6.540 copies/ml. The maximum viral load of 27,442 copies/ml was seen in one of the cases samples while the least viral of quantities 92 copies/ml was seen in one of the control samples (Fig. 3). The cases group have a viral load mean 6733 ± 6745 copies/ml which is significantly higher than that in the controls group 509.0 ± 792.9 copies/ml, with *P*-value of 0.002 (Table 2).

**Fig. 4:**
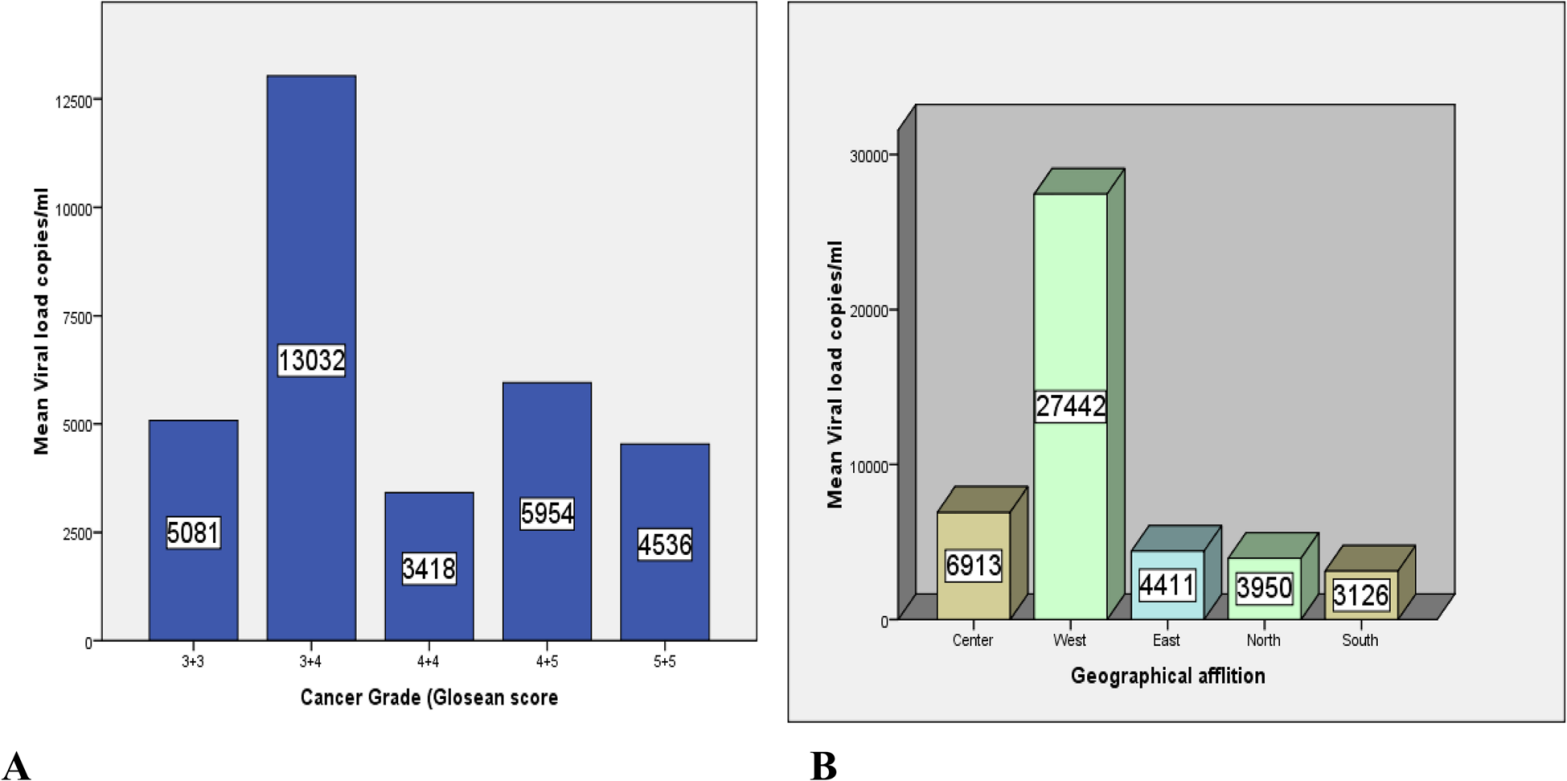
Prostate Cancer grades as well as case geographical affiliation versus BKV DNA load. **a** Viral quantities plotted against cancer grades. **b** viral load means plotted against the cases geographical affiliation

In the Pica cases group, the highest viral load mean was seen in patients with prostate cancer Gleason score of 7 (3 + 4) followed by score 9 (4 + 5), 6 (3 + 3), 10 (5 + 5) with viral load means of 13,032, 5954, 5081, 4536 copies/ml respectively. However, the least viral quantities of 3418 copies/ml were determined in patients with Gleason score 8 (4 + 4). These difference in the viral load means of the PCa grades was analyzed by ANOVA test and found that the cancer grades did not affect the viral load means with *P*-value of 0.489 (Fig. 4a).

In the PCa cases group, patients over the age of 65 years had a viral load mean of 6711.7 copies/ml which is slightly lower than those under the age of 656,786.8 copies/ml. Since the *P*-value was 0.561, the difference was negligible **(**Table 3**)*.*** By contrast, cases that expressed early symptoms reconditions durations for more than 9 months showed significantly higher viral quantities than those with less than 9 months with P-value of 0,045. **(**Table 3**)*.***

The result showed that the highest viral quantities were seen in Western Sudanese followed by Central, Eastern, Northern and Southern Sudanese with viral load mean of 27,442, 6913, 4411, 3950 and 3126 copies/ml respectively (Fig. 4b).

The effect of the environmental factors on *BK* viral load was analyzed by independent T-test and the result shows that alcohol consumption had a significant effect on viral quantities with P-value of 0.038. While other factors including working in the farmer, Diet high in fat, working in a tire plant and family history of cancer did not play an important role in the viral quantities (Table 7).

**Table 7:** Describe the effect of PC risk factors on BKV DNA Load. P-value is significant at a level equal to or less than 0.05

	Alcohol consumption		*P*-value
	Yes	NO	
Viral load mean Copies/ml	3135.29	9252.60	0.038
	Working in farmer		*P*-value
	Yes	No	
Viral load mean Copies/ml	9818.17	5051.27	0.310
	Diet high in Fat		*P*-value
	Yes	No	
Viral load mean Copies/ml	8156.25	5469.22	0.430
	Tire Plant Working		*P*-value
	Yes	No	
Viral load mean Copies/ml	248.00	7593.67	0.156
	Family history of the disease		*P*-value
	Yes	No	
Viral load mean Copies/ml	4534.33	7933.36	0.337

## Discussion

Prostate cancer (PCa) is a global health problem. It is estimated that there will be almost 1.3 million new cases of prostate cancer and 359,000 associated deaths worldwide in 2018, ranking second among the most frequent cancer cases and the fifth leading cause of cancer deaths among men [4]. Many studies have been conducted in different areas of PCa including causes, risk factors, and diagnosis and treatment options [27].

This study was conducted to determine the contribution of *BKV* infection and other environmental and lifestyle factors in the development of PCa. However, our study differed from its predecessors in two main points; the first in which we use the immune fluorescence technique to detect the *BKV* Large T antigen in the prostate tissue, the second point was that our study was first conducted in Sudan which located in Africa where there was little data available on the subject study with presence of high mortality rate due to prostate cancer [5].

Most previous studies have relied only on molecular techniques including conventional PCR and real-time PCR to determine the correlation between the PCa and *BKV* infection. Instead, our study is based on both detections of viral antigen via immunofluorescence technique and the confirmation of result by the molecular techniques including the conventional PCR and the real time PCR. The IF test result showed that 17 out of 55 cases were positive for LTAg while only 4 out of 55 controls subject were positive for the same antigen with a *P*-value of 0.002 and odd ratio 5.7 which mean that patients with this antigen in prostate tissues have a 5.7 risk of developing PCa of those who lack the same antigen in the prostate tissues. This high frequency detection of the LTAg in tissue samples of cases compared with controls is very strong evidence of the real virus contribution in PCa development rather than detecting the viral gene itself by PCR [28] because the active viral replication will produce the LTAg in the prostate tissue cells which will then bind the tumor suppresser antigens e.g. P53 resulted in disruption of apoptotic mechanism of the cell which subsequently leads to uncontrolled proliferation of prostate epithelia [29]. Our finding was agreed with that obtained by Das et al in 2008 who reported that the percentage of LTAg was significantly higher in cancerous prostates than in normal prostates But, they use Immunohistochemistry instead of the IF test [30, 31].

*BKV Large* T antigen could be supported by other factors such as age which may increase the carcinogenesis efficacy of the viral antigen. The current study found that those who were positive for the *BKV* antigen had age mean higher than those negative and that the viral antigen was also more frequently seen in cases over the age of 65 years than those under the age of 65. Even though the above difference was statistically insignificant we cannot neglect the high *BKV* infectivity rate among eldest patients with PCa in compared to those youngest. This leads us to suggest that aging can be a very important cofactor to the viral antigen in causing the development of PCa and this statement may need to be verified in further studies. Moreover, patients from central Sudan are more likely to develop PCa due to *BKV* compared to patients from other regions of Sudan and thus the geographical location can also be considered as another important *BKV* LTAg cofactor.

The current study also examined the effect of other factors such as alcohol consumptions, working in a farmer, taking a diet high in fat, working in a tire plant, and family history of cancer and cadmium contact on the efficacy of the LTAg in causing PCa and found it had no significant effect on the prevalence of *BKV* LTAg. Therefore, we concluded that the presence of antigen itself may be enough to develop PCa even in the absence of environmental cofactors and this was in agreement with Das et al finding in 2008 [30].

In our study, we calculate the DoPESR from the onset of acute PCa symptoms. We recognized that patients with DoPESR less than 9 months were more likely to be associated with a high prevalence of *BKV* LTAg than those with DoPESR more than 9 months. Therefore, we suggest that PCa can be developed due to the *BKV* is more aggressive and symptoms may develop earlier than PCa which has evolved for other reasons. Moreover, our study reported that the lowest *BKV* LTAg prevalence was seen in a patient with Gleason score 10. While Gleason score 7 and 9 had the highest *BKV* LTAg prevalence. This may be indicative for the lacking of the correlation between the cancer grades and *BKV* LTAg prevalence.

In this study, the samples that were given positive IF result were fatherly examined by using conventional PCR and real-time PCR. Using a conventional PCR, we found that 16 Out of 17 cases tissue samples were positive for the virus gene and only one was negative. In contrast 2 of 4 controls tissue samples were positive. Moreover, 4 urine samples out of 21 were positive for *BKV* gene of which only one urine sample was obtained from cases patients and the rest were from controls patients. From the above findings, the study here again reported very strong additional evidence in the involvement of the virus in developing PCa. The first point was high-frequency detection of the *BKV* DNA in the tissue of PCa patients compared with controls. The second is the nearly absence of the viral DNA in the cases urine samples suggesting that patients’ prostate tissue was the sole source of the virus. The third point was the presence of the *BKV* DNA in three out of four urine samples for controls patients meaning that the virus source was control patients urinary system and the virus may accidentally inhibit the prostate tissue.

During the last 20 years, a lot of molecular-based studies were conducted to determine the correlation between *BKV* infection and the development of PCa. Some of these studies accepted the hypothesis while others rejected this idea. In a study by Monini et al. (1995), in Italy, the *BKV* DNA was detected in more than 50% of both normal and tumor tissues obtained from the urinary tract and prostate using both PCR and Southern blotting. Moreover, they reported that the neoplastic tissues showed a significantly higher viral DNA load as compared to non-neoplastic tissues [32]. Since that report, five groups have examined prostate tissue for the presence of *BKV*. In 2002 Zambrano et al. in California (the USA) examined paraffin-embedded and fresh frozen tissue using conventional PCR. Although their results from fixed tissue were inconsistent, however, they detected *BKV* DNA in 25% of the frozen tissue [33]. Another report in Michigan (the USA) which done by Russo et al in 2008 detected *BKV* DNA in 85% of the PCa specimens but in none of the BPH control group by PCR and, hence, also suggests the role of *BKV* in the pathogenesis of PCa [34].. The most recent study which conducted in 2018 in Iran by Maryam et al.*,* reported that the viral DNA was identified in 9 patients (15%) with BPH compared to 17 patients of prostate cancer [35]. Our study thus gives additional support to the above findings. However, we apply the IF test to detect antigen responsible for the carcinogenesis and we confirm the result by using both conventional and real time PCR, while most previous studies have used only conventional PCR to investigate the hypothesis. The rationale for using both IF and molecular techniques in our study is that the virus nuclear material could be present in the cell as dormant (inactive) and does not produce an antigen. Therefore, it is important to detect the antigen by IF and confirm by molecular technique because this antigen can also be produced by a virus related to *BKV* such as *JC* and *SV40*. Also the presence of LTAg could be enough to prove the contribution of the virus to cause cancerous changes, because a lot of studies were proved the carcinogenic activity of the viral LTAg [21–29].

On the other hand, there are many examples of studies conducted in different countries in the world rejecting the hypothesis of the correlation between PCa and *BKV* infection. For instance the study by Lau et al. who used in situ hybridization (ISH) and Immunohistochemistry (IHC) to examine 30 cancerous prostate tissues. While they did not observe positive LTAg expression by IHC in any of their samples, their ISH results detected 4 samples containing *BKV* DNA in non-neoplastic cells, 2 samples in neoplastic cells [36]. Another study conducted by Sfanos et al in 2008 analyzed a total of 200 patients for the presence of *BKV* DNA using nested PCR. Surprisingly, only one sample was positive for *BKV* [37]. A study was done by Groom et al.*,* on a 100 PCa tissue samples tested for *BKV* DNA by nucleic acid detection techniques was failed to detect the presence of *BKV* DNA in all samples even though DNA integrity and assay sensitivity have been demonstrated [38].

Only few studies have detected the viral DNA in urine samples of PCa patients. A study done by Monica in 2011 detected the virus DNA in the urine of 14/26 (54%) cases while she didn’t find any viral evidence within urine samples from control subjects [39]. This finding has severely disagreed with our finding and this can be explained by the fact that she used a real-time for viral detection in the urine samples instead, we use the conventional PCR for detection of viral DNA prior to quantitation measurement.

It’s worth to mention that the only sample that gives a positive IF test for LTAg and reveals a negative result when analyzed later by conventional PCR and Real-time can be classified as a *JC virus* that containing about 75% shared genome homology with *BKV*. Since there is a similarity in the genome, it may result in amino acids sequence similarity leading to cross-reaction during IF test. This can be demonstrated by detecting JCV DNA in this sample of the patient [16].

Real-time PCR may be superior to the conventional PCR in the sensitivity of the assay. Since the virus may appear latent as a part of an infected cell genome, the detection of the viral load together with antigen expression may be more useful in clarifying the cancer-causing activity of the virus. When we examined tissue samples by real-time PCR we found a highly significant difference between cases and control of their viral load means. The cases group showed a viral load means of 6733 ± 6745 copies/ml which is higher than that of control subjects’ 509.0 ± 792.9 copies/ml. This is also another import proves in the contribution of the virus in developing PCa. Although the importance of real-time PCR in viral load analysis, it has been used only in a few previous studies. For instance, four previous studies were used a real-time PCR to analyze the correlation between the *BKV* and PCa and found that out of a total of 482 PCa patients the prevalence rate of the virus was around 20% in their tissue samples [40]. The results were consistent with our findings where the prevalence of BKV between our study cases was 30% by the IF test and 94% by PCR in real-time.

In this study, the highest viral load was seen in cases patients with PCa Gleason score 7 (3 + 4) followed by score 9 (4 + 5), 6 (3 + 3), 10 (5 + 5) with viral load means of 13,032, 5954, 5081, 4536 copies/ml, respectively. However, the least viral quantities of 3418 copies/ml were seen in patients with Gleason score 8 (4 + 4). This difference in the viral load means for the PCa grades was analyzed by ANOVA test and found that the cancer grades did not affect the viral load means with *P*-value of 0.489. However, in a study conducted in Italy by Monica in 2011 she measured the viral load of *BKV* among PCa patients and she found that the highest viral load mean was seen between patients with Gleason score 9 (16,914) whereas Gleason 8 patients show an average of 13,300 cells and Gleason 7 showed an average of 9457 [39].

In the current study, the age and DoPESR were analyzed across the viral load and we found that in the cases group, patients over the age of 65 years had a viral load mean of 6711.7 copies/ml which is slightly lower than those under the age of 656,786.8 copies/ml. Since the P-value was 0.561, the difference was negligible. By contrast, cases that experienced symptoms for more than 9 months showed significantly higher viral quantities than those less than 9 months with *P*-value of 0.045. The latter finding can be explained by a longer DoPESR time giving a greater chance for virus replication.

The study also determined the effect of the patients’ geographical affiliation on their own viral load and found that the highest viral quantities were seen in samples of Western Sudanese followed by Central, Eastern, Northern and Southern Sudanese samples with viral load mean of 27,442, 6913, 4411, 3950 and 3126 copies/ml, respectively. This high virus replication rate in Western Sudanese samples may be associated with certain environmental factors such as lack of healthy water intake [41]. The situation in Darfur (Western Sudan) is considerably worse than in other states in Sudan; only 26% of the population has access to an improved water source compared to an average of 64% in other rural areas of the country [41]. In addition to environmental factors, nutritional factors may also play a critical role. Conflict in the Western parts of Sudan compounded by climatic problems such as drought and floods have caused severe food deficits, loss of livelihoods and major population displacements [41].

Furthermore, the effect of other factors such as alcohol consumption, working in the farmer, diet high in fat, working in a tire plant and family history of cancer on the viral quantities were analyzed. Alcohol consumption was found only to have a significant effect on the viral quantities and thus the viral replication (*P*-value 0.038). While the remaining factors showed an insignificant effect on viral replication. Why alcohol consumption over all other studied factors? Because it usually affects the liver and thus the general body health which gives a great opportunity to virus replication [42]. Therefore, alcoholism may increase the risk to develop PCa due to *BKV* Infection.

At the end of the discussion, we would like to note an important epidemiological observation. Regarding the frequency of Gleason cores among the cases group we determine a high agreement between our finding and those of Taghavi et al.*,*. Our study reported that among cases group the Gleason score 7 (3 + 4) was the most identified PCa grade followed by score 6 (3 + 3), 9(4 + 5), 8(4 + 4) and 10 (5 + 5) with a percentage of 34.5, 21.8, 20.0, 18.2, 5.5% respectively and Taghavi et al.*,* in Iran 2015 were also found that Gleason score 7 was most common followed by score 6,9,8,10 and 2 with a percentage of 33%, %22, 18, 15, 8, and 3% respectively [43]. This observation may need further investigation from those who are interested in PCa epidemiological studies.

Our study has some limitations, unfortunately, we did not receive any kind of funding. Therefore not all cases and controls samples (110) were tested with both IF and qRT-PCR collectively. We use firstly the IF assay followed by PCR analysis. In the future studies, we suggest analyzing all samples by the both above mentioned technique this may yield a much more reliable result.

## Conclusion

We concluded that more *BKV* LTAg prevalence with high viral load was observed in PCa patients’ tissue specimens compared to BPH specimens. The viral DNA was detected in only one urine sample of cases patients. Also, *BKV* infection was most predominantly observed in cancerous specimens with total Gleason scores 9 and 7. However, Gleason score 7 was associated with the highest viral load. In addition, among cases group, Central Sudanese were the most affected group with both PCa and *BKV* infection while Western Sudanese were associated with the highest viral quantities. Furthermore, alcoholism may increase the risk to develop PCa due to *BKV* Infection. Our findings are thus consistent with a significant link between the *BK V* infection and the PCa risk.

It is highly recommended to use the multiplex IF assay to detect both viral LTAg and P53 antigen which is an apoptotic signal in the future studies. Moreover, the using of real-time PCR to measure the quantities of the virus LTAg gene mRNA is also recommended. Cell culture technique is highly recommended in the future studies because it can help the researcher to monitor the quantities and interaction of the *BKV LTAg* that produced by the virus and bind to the cell tumor suppresser antigens. Furthermore, Prostate or seminal fluids should be selected as principal specimens for future studies and can therefore, be designated as screening samples to find early virus evidence in the prostate tissue. Detection of early virus evidence may help to reduce the risk of cancer due to BKV.

## Data Availability

The datasets used and/or analyzed during the current study are available from the corresponding author on reasonable request.

## Abbreviations

*BKV*: *BK Virus*
BPH: Benign prostatic hyperplasia
DAF: Direct immunofluorescence assay
DoPESR: Duration of patients early symptoms recognitions
IF: immunofluorescence
IHC: Immunohistochemistry
ISH: insituhyperdyzation
LTAg: large tumor antigen
PCa: prostate cancer
Q-PCR: quantitative PCR
R-PCR: Real-time PCR
STAg: small tumor antigen
